# Measurement Properties and Minimal Detectable Change of the 6‐Min Step Test for Estimating Cardiorespiratory Fitness in Hospitalized Individuals After Stroke

**DOI:** 10.1002/pri.70201

**Published:** 2026-03-26

**Authors:** Raissa Olegário Aguiar Pavesi, Augusto Boening, Silvana dos Santos Meyrelles, Lucas R. Nascimento

**Affiliations:** ^1^ Department of Physiotherapy Universidade Federal Do Espírito Santo Vitória Brazil; ^2^ Department of Physiological Sciences Universidade Federal Do Espírito Santo Vitória Brazil

**Keywords:** aerobic, cardiorespiratory fitness, cerebrovascular accident, submaximal test

## Abstract

**Background:**

The 6‐minuteute step test is an alternative for estimating cardiorespiratory fitness after stroke.

**Objective:**

To investigate its measurement properties and minimal detectable change early after stroke.

**Methods:**

Cross‐sectional, methodological study. Participants were inpatient individuals in the acute/subacute phase after stroke, who performed the 6‐min step and the 6‐min walk tests. The outcomes of interest were test–retest and inter‐rater reliabilities, validity, measurement error, and minimal detectable change.

**Results:**

Fifty‐one individuals (34 men) with a mean time of 6 days (SD 2) since stroke, and a mean walking speed of 0.9 m/s (SD 0.2) were included. The 6‐min step test had a very‐high test‐retest (ICC 0.93; 95% CI 0.88–0.96) and inter‐rater reliability (ICC 0.95; 95% CI 0.91–0.97). The correlation between the 6‐min‐step test and the 6‐min walk was moderate (*r* = 0.5; 95% CI 0.3–0.7). The measurement error was 8 (10%) and the minimal detectable change was 21 repetitions.

**Conclusion:**

The 6‐min step test demonstrated appropriate test‐retest and inter‐rater reliability. Agreement with the 6‐min walk test was imprecise, which may reflect the greater physical demand of the 6‐min‐step test. Further studies are needed to examine the relationship between the 6‐min‐step test and maximal cardiorespiratory capacity.

## 1 Introduction

People after stroke commonly experience a marked decline in cardiorespiratory fitness, which is particularly evident in the early stages of recovery (Oyake et al. 2017; Smith et al. 2012). Lower levels of cardiorespiratory fitness are associated with greater functional disability (*r* = 0.50; *p* = 0.03) and increased limitations in daily activities (*r* = 0.50; *p* = 0.02) (Kossi et al. 2024; Larsson et al. 2024). Clinical guidelines emphasize the importance of initiating cardiovascular training early, including during hospitalization (Hebert et al. 2016; Billinger et al. 2014; MacKay‐Lyons et al. 2020). This recommendation highlights the need for appropriate measurement of cardiorespiratory fitness in clinical practice. While the cardiopulmonary exercise test is the gold standard (American Thoracic Society 2002), its use is often limited by cost and by the clinical instability and motor impairments frequently present in the acute phase after stroke (Di Thommazo‐Luporini et al. 2012; Richards et al. 1999). For these reasons, clinicians commonly adopt the 6‐min walk test as an alternative (Di Thommazo‐Luporini et al. 2012). However, the requirement for a long and unobstructed corridor restricts its feasibility in many rehabilitation environments (Fell et al. 2022).

The 6‐min‐step test is an alternative to evaluate cardiorespiratory fitness. It is a submaximal test that requires minimal equipment and space, making it feasible for use in hospital settings (Pessoa et al. 2014; Bisca et al. 2015). This test has been applied in populations with cardiac and pulmonary conditions and has demonstrated adequate measurement properties, including very high test–retest reliability (ICC = 0.96; 95% CI 0.91 to 0.98; *n* = 125), high inter‐rater reliability (ICC = 0.80; 95% CI 0.62 to 0.89; *n* = 32), and moderate criterion validity when compared with maximal exercise testing (*r* = 0.53; 95% CI 0.30 to 0.71; *n* = 307) (Boening et al. 2024; Gagnier et al. 2021). In people after stroke, the test has also shown high test–retest reliability (ICC = 0.98; 95% CI 0.97–0.99), high inter‐rater reliability (ICC = 0.95; 95% CI 0.92–0.97), and good construct validity with the 6‐min walk test (*r* = 0.80; 95% CI 0.70–0.90) (Boening, Aguiar, et al. 2025). However, these findings were obtained in individuals in the chronic phase who were not hospitalized, which limits the generalization of results to those in the acute or subacute phases, where clinical and functional conditions differ substantially. Therefore, evidence on the measurement properties of the 6‐min‐step test in hospitalized individuals in the early phase after stroke is still lacking.

In the early stages after stroke, individuals typically present greater clinical instability, which may increase the risk of adverse events and compromise the safe performance of exercise tests. Additionally, measurement properties are population dependent. Thus, a test that is reliable and valid in the chronic phase cannot be assumed to be appropriate for clinical evaluation in the acute or early subacute phases (Portney 2020). Therefore, the purpose of this study was to investigate the measurement properties of the 6‐min step test in inpatient individuals in the acute or early subacute phases after stroke, following standardized procedures. The specific research questions are.

1. Does the 6‐min step test demonstrate appropriate measurement properties (test–retest reliability, inter‐rater reliability, measurement error, and construct validity) for estimating cardiorespiratory fitness in individuals in the early stages after stroke?
2. What is the minimal detectable change of the test in this population?

## 2 Method

### 2.1 Design

This cross‐sectional methodological study was conducted in accordance with the COSMIN recommendations and reported according to the STARD recommendations (Mokkink et al. 2010). Ethical approval was obtained from the Institutional Research Ethics Committee (CAAE 59,441,422.30000.5060), and all participants provided written informed consent prior to data collection. The study took place at Hospital Estadual Central, the leading center for stroke treatment in a metropolitan city (Vitória, Espírito Santo, Brazil). The data collection period was from October 2024 to September 2025.

### 2.2 Participants

A sample size of 50 individuals was planned in accordance with the COSMIN standards for adequate sample size in studies evaluating measurement properties (Gagnier et al. 2021). Individuals in the acute and early subacute phases after stroke were screened through review of medical records and recruited by convenience sampling. Participants were included if they: (1) were aged 18 years or older; (2) were between one day and three months after stroke onset, corresponding to the acute and early subacute phases; (3) were inpatients still hospitalized for stroke care; (4) were able to walk with or without assistive devices, or had a Functional Ambulation Category score ≥ 3 (Sullivan et al. 2013; Nascimento et al. 2021); and (5) were able to step up and down a 20‐cm step. Participants were excluded if they: (1) were unable to understand and follow verbal commands; (2) presented any unstable health condition, identified by heart rate more than 120 bpm, systolic blood pressure > 180 mmHg, diastolic blood pressure more than 120 mmHg, pain, or edema before testing; or (3) had an orthopedic condition causing walking limitations.

The following clinical data were collected for characterization purposes: age, sex, type and time since stroke, walking speed, walking confidence, functional limitations, and dyspnea. Walking speed was measured using the 10‐m walk test, and reported in meters per second (m/s) (Cheng et al. 2020; Tyson and Connell 2009). Participants were instructed to walk at a “usual, safe and comfortable walking pace” (Nascimento et al. 2012) along a 14‐ m hallway, and the time to cover the central 10 m was recorded with a digital stopwatch. Walking confidence was measured using the Brazilian version of the Modified Gait Efficacy Scale, with scores ranging from 10 to 100 points, where higher scores represent greater confidence while walking (Avelino et al. 2022). Functional limitations were measured using the Modified Rankin Scale, with scores ranging from 0 to 6, where higher scores indicate greater dependence on performing everyday activities (Baggio et al. 2014). Dyspnea was measured using the Medical Research Council, with scores ranging from one (i.e., “I only get breathless with strenuous exercise” to five (i.e., “I am too breathless to leave the house”) (Bestall et al. 1999).

### 2.3 Outcomes

The outcomes of interest were the number of repetitions on the 6‐min step test (defined as one complete cycle of stepping up and down) and the distance covered during the 6‐min walk test (meters).

The 6‐min‐step test was performed using a 20‐cm‐high step positioned in front of a handrail. Participants were instructed to step up and down as quickly as possible for 6 minutes at their own pace. If necessary, participants could rest during the test without stopping the timer. Standardized verbal encouragement was given every minute (e.g., “You are doing well, keep going”) in a neutral tone of voice, and participants were also informed each minute of the remaining time. The total number of steps completed in 6 minutes was recorded using a manual analog counter (Boening et al. 2024; American Thoracic Society 2002). The 6‐min walk test was performed in a 30‐m corridor free of obstacles. Participants were instructed to walk as far as possible for 6 minutes at their own pace. If necessary, participants could rest during the test without stopping the timer. Standardized verbal encouragement was given every minute. The total distance walked in meters in 6 minutes was recorded (American Thoracic Society 2002).

Both tests were performed in the same location to ensure consistent testing conditions, in a hallway in the section where the participants were hospitalized, during periods of reduced traffic. Two assessors conducted the tests (assessor 1 and assessor 2). Assessor one is a physiotherapist currently undertaking a master's degree in neurological rehabilitation, and assessor two is a final‐year physiotherapy student. Clinical stability was ensured throughout the testing protocol. Physiological measurements (heart rate, blood pressure, and oxygen saturation) were monitored before, immediately after, and 2 minutes following each test. Exertion fatigue was measured immediately after testing using the Borg scale, ranging from 0 (no fatigue) to 10 (maximum fatigue) (Tseng et al. 2010). Tests could be interrupted in the event of severe adverse reactions, including chest pain, intolerable dyspnea, leg cramps, staggering, diaphoresis, falls, loss of coordination, mental confusion, or pallor (American Thoracic Society 2002). Tests were performed only when participants were clinically stable and vital signs had returned to baseline. In unstable situations, the healthcare team was notified.

The full protocol consisted of three 6‐min‐step tests and one 6‐min walk test, with a 30‐min rest interval between tests. First, two 6‐min‐step tests were performed by assessors 1 and two in random order. Then, the 6‐min walk test was performed, followed by a third 6‐min step test conducted by assessor 1. The order of the step and walk tests was not randomized due to operational limitations imposed by hospital routine and technical issues related to protocol execution. This approach reflects real clinical practice conditions. The duration and timing of the protocol varied according to hospital schedules (e.g., examinations, medications, meals, and hallway traffic). Consistent testing conditions, prior familiarization, and standardized rest intervals were used to minimize order, learning, and fatigue effects. Participants were informed of their results only after completion of the entire protocol to minimize potential bias.

### 2.4 Measurements Properties

The examined measurement properties were test‐retest reliability, inter‐rater reliability, measurement error, and construct validity. In addition, the minimal detectable change in the test was calculated. Detailed procedures are as follows: Two trials of the 6‐min step test were performed by the same therapist to assess test‐retest reliability, and a third trial was performed by a different therapist to assess inter‐rater reliability. Test‐retest reliability was defined as the ability of the 6‐min step test to reproduce similar results (number of steps) when performed at different times by the same therapist. Inter‐rater reliability was defined as the ability of the test to reproduce similar results when performed by a different therapist (Souza et al. 2017). Measurement error was defined as the error of a patient's score not attributed to true changes in the measured construct, and was calculated using the standard error of measurement (SEM) based on test‐retest data (Portney 2020). Minimal detectable change was defined as the amount of change required to ensure that observed differences exceeded measurement error (Portney 2020). Construct validity was defined as the ability of the 6‐min step test to provide accurate information in comparison with the most common clinical submaximal test (i.e., 6‐min walk test) (Souza et al. 2017).

### 2.5 Analyses

Descriptive statistics were used to characterize the sample. Data normality was examined by the (Kolmogorov‐Smirnov). Test‐retest and inter‐rater reliability were assessed using the intraclass correlation coefficient (ICC) based on a 2‐way random effects model, single rater, absolute agreement, and reported with respective confidence intervals (CI). This model quantifies absolute agreement by partitioning variance attributable to participants, raters, and residual error, allowing detection of both random and systematic measurement errors (Portney 2020; Gagnier et al. 2021). A paired *t*‐test was performed to examine potential systematic differences between repeated measurements of the 6‐min step tests, while agreement was performed using Bland–Altman analysis to assess systematic bias across the range of measurements (Portney 2020; Gagnier et al. 2021). Comparisons between the 6‐min step test and 6‐min walk test were evaluated using Pearson's correlation coefficient, given the normal distribution of the data, and reported with respective CIs. The magnitude of the results was classified as: very high (0.90–1.00), high (0.70–0.89), moderate (0.50–0.69), low (0.26–0.49), and very low (0–0.25) (Munro 2005). The measurement error was calculated using the standard error of the measurement (SEM) as follows: SEM = s√(1.00 – *r*), where *s* is the pooled standard deviation of the test‐retest measurements and *r* is the test‐retest ICC (Tseng et al. 2010). The minimal detectable change (MDC) was calculated at the individual level using a 95% CI, according to the formula: MDC = 1.96*SEM*√2 (Portney et al., 2020).

The relative SEM and MDC were also calculated, to guarantee the proportion or error and change presented in the sample. The SEM% and the MDC% were calculated as follows: SEM% = (SEM/mean)*100, and MDC% (MDC/mean)*100, respectively, where the mean is the number of repetitions performed by participants (Cheng et al. 2020; Flansbjer et al. 2005). A SEM < 10% of the range score was considered small (Quintino et al. 2021).

## 3 Results

**FIGURE 1:**
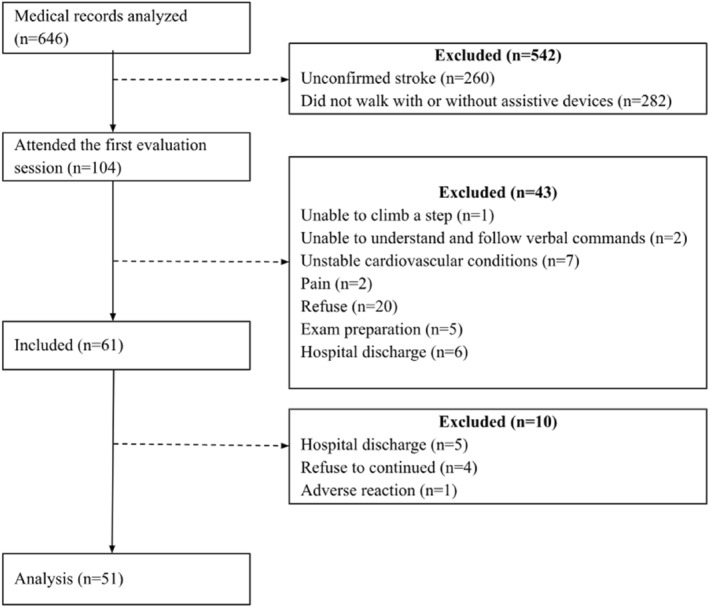
Flowchart of participants.

A total of 646 individuals were screened; however, 542 were not included due to unconfirmed diagnosis of stroke (*n* = 260; 40%) or inability to walk (*n* = 282; 44%). As a result, 104 participants attended the first evaluation session, but 43 did not meet the inclusion criteria (see Figure 1 for details). Therefore, 61 individuals were initially included. During the testing procedures, 10 participants were excluded due to hospital discharge (*n* = 5), refusal to continue (*n* = 4), or an adverse reaction (mental confusion; *n* = 1). Thus, 51 individuals (34 men), with a mean age of 61 years (SD 13) and a mean time after stroke of 6 days (SD 2), were included in the final analysis. In addition, seven participants (14%) used assistive devices to perform the 6‐min walk test. Their descriptive data are summarized in Table 1. Overall, participants presented relatively mild neurological impairment, as indicated by a mean Modified Rankin Scale score of 1.5 and a mean walking speed of 0.9 m/s.

**TABLE 1:** Participants' characteristics.

Variable	*n* = 51
Age (years), mean (SD)	61 (13)
Sex, *n* male (%)	34 (66)
Time since stroke (days), mean (SD)	6 (2)
Type of stroke, *n* ischemic (%)	47 (92)
Previous stroke, yes (%)	10 (20)
Walking speed (m/s) (10mWT), mean (SD)	0.9 (0.2)
Walking confidence (mGES) (0–100), mean (SD)	73 (18)
Functional limitations (mRS) (0‐6), mean (SD)	1.5 (1.2)
Dyspnea (mRC) (1‐5), mean (SD)	1.2 (0.6)
Number of repetitions (6‐min step test), mean (SD)	78 (30)
Walking distance (m) (6‐min walk test), mean (SD)	342 (92)

Abbreviations: 10mWT = 10‐m walking test; mGES = Modified Gait Efficacy Scale; mRC = Medical Research Council; mRS = Modified Rankin Scale; SD = Standard deviation.

Overall, participants stepped up and down 78 times (SD 30), ranging from 21 to 145 repetitions, during the 6‐min step test, and most participants (55%) performed less than 78 repetitions. Participants walked an average of 342 m (SD 92) during the 6‐min walk test. The full protocol was completed in a mean of 1 day (SD 0.3); however, six participants (12%) completed it over 2 days. The number of participants who required a rest period and rest duration is provided in Table 2. Fatigue was the main reason for resting during the 6‐min step test (*n* = 29; 88%), whereas lower‐limb pain was the main reason in the 6‐min walk test (*n* = 2; 100%). All participants completed the tests, and no adverse events occurred.

**TABLE 2:** Number of participants (%) who required a rest period and rest duration (seconds).

Variable	*n* = 51
First 6‐min step test	
Rest, *n* yes (%)	10 (5)
Rest duration (s), mean (SD)	58 (47)
Second 6‐min step test	
Rest, *n* yes (%)	11 (6)
Rest duration (s), mean (SD)	53 (48)
Third 6‐min step test	
Rest, *n* yes (%)	12 (6)
Rest duration (s), mean (SD)	60 (38)
First 6‐min walk test	
Rest, *n* yes (%)	2 (1)
Rest duration (s), mean (SD)	82 (0.7)

Abbreviations: s = seconds; SD = Standard deviation.

**FIGURE 2:**
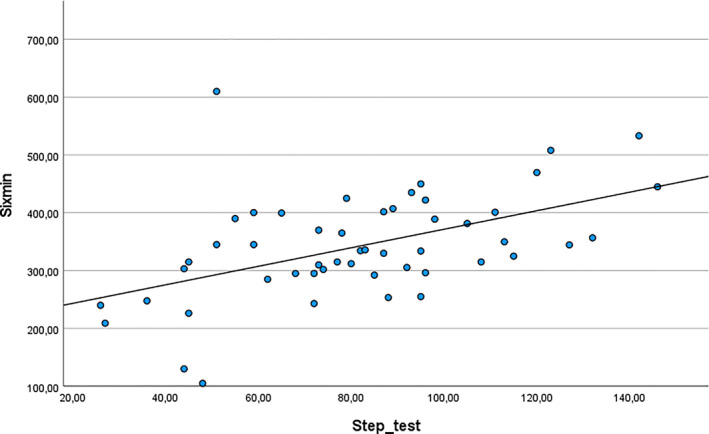
X‐axis = 6‐min step test (repetitions); y‐axis = 6‐min walk test (meters).

The 6‐min step test had very high test‐retest reliability (ICC 0.93; 95% CI 0.88–0.96), and very high inter‐rater reliability (ICC 0.95; 95% CI 0.91–0.97). Test‐retest reliability was supported by a paired *t*‐test showing no significant difference between repeated measurements performed by the same examiner (MD −2 repetitions; 95% CI −6 to 2), and by Bland‐Altman analysis showing minimal systematic bias (MD −2 repetitions; 95% CI −6 to 2). Inter‐rater reliability was supported by a paired *t*‐test showing no significant difference between measurements performed by different examiners (MD 1 repetition; 95% CI −3–5), and by Bland‐Altman analysis showing minimal systematic bias (MD 1 repetition; 95% CI −3–5). The measurement error (standard error of the measurement) was 8 repetitions (10%). The correlation between the 6‐min step test and the 6‐min walk test indicated a moderate construct validity (*r* = 0.5; 95% CI 0.3–0.7) (Figure 2). The minimal detectable change was 21 repetitions. Detailed results are provided in Table 3.

**TABLE 3:** Measurements properties of the 6‐min‐step test early after stroke.

Variable	*n* = 51
Test‐retest reliability, ICC (95% CI)	0.93 (0.88–0.96)
Inter‐rater reliability, ICC (95% CI)	0.95 (0.91–0.97)
Measurement error, SEM (SEM%)	8 (10)
Minimal detectable change, MDC (MDC%)	21 (27)
Construct validity, *r* (95% CI)	0.5 (0.3–0.7)

Abbreviations: CI = Confidence interval; ICC = Intraclass correlation coefficient; MDC = minimal detectable change; *r* = Pearson's correlations; SEM = Standard error measurement.

## 4 Discussion

The 6‐min step test showed very high test–retest (ICC = 0.93) and inter‐rater (ICC = 0.95) reliabilities, along with an acceptable measurement error (SEM = 8 repetitions; 10%) and a minimal detectable change of 21 repetitions in hospitalized individuals early after stroke. The test also showed moderate construct validity when compared with the 6‐min walk test (*r* = 0.50), suggesting that the two tests capture related but not identical aspects of functional capacity. Additionally, the test appeared safe and feasible for estimating cardiorespiratory fitness in hospitalized patients with mild limitations early after stroke, with high completion rates in the first week of recovery.

In the present study, the participants performed, on average, 78 repetitions during the 6‐min step test, ranging from 21 to 145 repetitions, which indicates appropriate variability for the examination of measurement properties. Most participants (55%) performed fewer than 78 repetitions, which is an indicator of poor cardiorespiratory fitness (Pessoa et al. 2014). This was similar, in part, to the previous study (Boening, Aguiar, et al. 2025), which found that individuals in the chronic phase performed an average of 88 repetitions (*n* = 50); however, most participants (60%) surpassed the threshold of 78 repetitions. Taken together, these results indicate that the 6‐min step test captures meaningful variability across different stages of stroke recovery, which aligns with evidence showing that cardiorespiratory fitness changes over time (Oyake et al. 2017; Smith et al. 2012). For instance, a systematic review (Smith et al. 2012) demonstrated lower levels in the early stages and an increase of approximately 15% over the first 6 months. It is also important to acknowledge that patients with more severe limitations are unlikely to be able to step onto a 20‐cm platform and therefore would not be eligible for this assessment, meaning that cardiorespiratory fitness in the acute/subacute phases may appear higher than it truly is for the broader inpatient population. In this context, the performance observed in our sample likely reflects their clinical profile, as they had mild activity limitations, demonstrated by an average walking speed of 0.9 m/s. This level of functionality supports their ability to engage in submaximal step‐based exercise tests and may help explain the relatively preserved cardiorespiratory fitness observed in the first week after stroke.

The analyses demonstrated very high test–retest and inter‐rater reliability with narrow confidence intervals, which suggests that the results are credible, similar to findings reported in individuals in the chronic phase (Boening, Aguiar, et al. 2025). These results indicate that the 6‐min‐step test can reproduce similar results with consistency when performed at different times by the same or different therapists. In addition, the measurement error of 8 repetitions can be classified as appropriate (10%) (Huang et al. 2011; Green et al. 2002). On the other hand, the minimal detectable change was 21 repetitions (27%) (Huang et al. 2011; Green et al. 2002). This means that variations above or below this value reflect real changes in step‐climbing fitness over time. However, this also indicates that the test is not sensitive to small improvements, and its results cannot be directly converted into physiological measures such as VO_2_. From a clinical perspective, this means that a patient with low to moderate cardiorespiratory fitness who performs around 60 repetitions would need an improvement of approximately 40% before being clearly identified as having good cardiorespiratory fitness, that is, surpassing the threshold of 78 repetitions.

This study also showed that the 6‐min step test is correlated with the 6‐min walk test; however, the magnitude of the correlation was moderate, with wide confidence intervals, which indicates imprecision. This finding may reflect the greater physiological demand of the step test than the 6‐min walk test. Therefore, although related, the two tests may reflect different physiological demands during functional exercise after stroke. For instance, in the present study, fatigue was the main reason for resting during the 6‐min step test (*n* = 29; 88%), whereas no fatigue‐related rest was observed during the 6‐min walk test. This pattern is consistent with evidence that stair climbing requires greater oxygen consumption (VO_2_ 1.1 L/min; SD 0.3) than walking (VO_2_ 0.6 L/min; SD 0.2) after stroke (Polese et al. 2018). This hypothesis has been partially supported by an experimental study showing that the 6‐min step test elicited higher dyspnea (MD 1 point out of 10; 95% CI 0.5–2) and leg fatigue (MD 2 points out of 10; 95% CI 1–2) immediately after testing. In addition, heart rate was consistently higher during the 6‐min step test (by 7–15 bpm), and remained higher immediately afterward (MD 17 bpm; 95% CI 11–22), in a sample of 57 individuals with stroke (Boening, Aguiar, et al. 2025).

To our knowledge, this is the first study to investigate an alternative submaximal test for estimating cardiorespiratory fitness in hospitalized patients during the acute/subacute phases after stroke. However, it was not without limitations. First, the sample was not randomly selected and included ambulatory individuals able to step onto a 20‐cm platform, which may limit the extent to which the findings generalize to the broader acute/subacute stroke population, particularly to patients with more severe impairments. Second, the test order was not randomized; although procedures were standardized, learning or fatigue effects may have influenced the results and represent a potential source of bias. Finally, oxygen consumption was not directly measured, which prevented establishing criterion validity against a gold‐standard cardiopulmonary test. Given that no adverse events occurred and the test appeared safe and feasible early after stroke, an important next step would be to evaluate its performance in both acute and chronic patients while directly comparing it with a maximal cardiopulmonary exercise test.

In conclusion, the 6‐min‐step test can be safely administered to hospitalized patients in the early stages of stroke. It demonstrated appropriate test‐retest and inter‐rater reliability; however, its agreement with the 6‐min walk test was imprecise, indicating that the two tests should not be used interchangeably. Further studies should examine the relationship between the 6‐min‐step test and maximal cardiorespiratory tests to better establish its validity.

## 5 Implications of Physiotherapy Practice

- The 6‐min‐step test is a reliable and feasible option for estimating cardiorespiratory fitness in hospitalized individuals early after stroke.
- The test requires minimal equipment and space requirements, making it practical for use in hospital environments where corridor‐based tests may not be possible.
- The minimal detectable change is 21 repetitions, which helps clinicians interpret true improvements over time.
- The test may offer a more demanding alternative to walking‐based assessments, potentially capturing meaningful changes in fitness during early rehabilitation.

## Funding

Funding from the Brazilian Government Funding Agencies: Coordenação de Aperfeiçoamento de Pessoal de Nível Superior (CAPES) ‐ Finance Code 001, Fundação de Apoio à Pesquisa e Inovação do Espírito Santo (FAPES) (Grant 13/2025), and Conselho Nacional de Desenvolvimento Científico e Tecnológico (CNPq) (Grant 406,375/2025‐9). The last author (LRN) received a Research Productivity Fellowship from FAPES.

## Ethics Statement

CAAE 59.441.422.30000.5060. UFES – 10/09/2024. All participants provided written consent prior to data collection.

## Consent

Written informed consent was obtained from all participants prior to data collection.

## Conflicts of Interest

The authors declare no conflicts of interest.

## Data Availability

The data that support the findings of this study are available from the corresponding author upon reasonable request.

## References

[pri70201-bib-0001] American Thoracic Society . 2002. “ATS Statement: Guidelines for the Six‐Minute Walk Test.” American Journal of Respiratory and Critical Care Medicine 166, no. 1: 111–117. 10.1164/ajrccm.166.1.at1102. 12091180

[pri70201-bib-0002] Avelino, P. R. , L. R. Nascimento , K. K. Menezes , M. T. M. Alvarenga , I. Faria Fortini , and L. F. Teixeira‐Salmela . 2022. “Test‐Retest Reliability and Measurement Error of the Modified Gait Efficacy Scale in Individuals With Stroke.” Physiotherapy Theory and Practice 38, no. 13: 2956–2961. 10.1080/09593985.2021.1952669. 34294003

[pri70201-bib-0003] Baggio, J. A. O. , T. E. G. Santos‐Pontelli , P. T. Cougo‐Pinto . 2014. “Validation of a Structured Interview for Telephone Assessment of the Modified Rankin Scale in Brazilian Stroke Patients.” Cerebrovascular Diseases 38, no. 4: 297–301. 10.1159/000367646. 25412853

[pri70201-bib-0004] Bestall, J. C. , E. A. Paul , R. Garrod , R. Garnham , P. W. Jones , and J. A. Wedzicha . 1999. “Usefulness of the Medical Research Council (MRC) Dyspnoea Scale as a Measure of Disability in Patients With Chronic Obstructive Pulmonary Disease.” Thorax 54, no. 7: 581–586. 10.1136/thx.54.7.581. 10377201 PMC1745516

[pri70201-bib-0005] Billinger, S. A. , R. Arena , J. Bernhardt , and Council on Clinical Cardiology . 2014. “Physical Activity and Exercise Recommendations for Stroke Survivors: A Statement for Healthcare Professionals From the American Heart Association/American Stroke Association.” Stroke 45, no. 8: 2532–2553. 10.1161/STR.0000000000000022. 24846875

[pri70201-bib-0006] Bisca, G. W. , A. A. Morita , N. A. Hernandes , V. S. Probst , and F. Pitta . 2015. “Simple Lower Limb Functional Tests in Patients With Chronic Obstructive Pulmonary Disease: A Systematic Review.” Archives of Physical Medicine and Rehabilitation 96, no. 12: 2221–2230. 10.1016/j.apmr.2015.07.017. 26254951

[pri70201-bib-0007] Boening, A. , L. T. Aguiar , J. Avance , and L. R. Nascimento . 2025. “The 6‐min Step Test Elicits Higher Physiological Responses Than the 6‐min Walk Test in People With Stroke: A Cross‐Sectional Study.” Journal of Bodywork and Movement Therapies 44: 802–807. 10.1016/j.jbmt.2025.07.013. 40954665

[pri70201-bib-0008] Boening, A. , A. A. Scianni , J. Avance , M. T. M. Alvarenga , and L. R. Nascimento . 2025. “Measurement Properties of the 6‐Min Step Test for Estimating Cardiorespiratory Fitness in Individuals With Chronic Stroke.” Topics in Stroke Rehabilitation 32, no. 8: 829–837. 10.1080/10749357.2025.2494963. 40270114

[pri70201-bib-0009] Boening, A. , A. A. Scianni , J. A. Martins , C. H. Santuzzi , F. M. Liberato , and L. R. Nascimento . 2024. “Procedures and Measurement Properties of the 6‐Min Step Test: A Systematic Review With Clinical Recommendations.” Clinical Rehabilitation 38, no. 5: 647–663. 10.1177/02692155241229286. 38311940

[pri70201-bib-0010] Cheng, D. K. , M. Nelson , D. Brooks , and N. M. Salbach . 2020. “Validation of Stroke‐Specific Protocols for the 10‐Meter Walk Test and 6‐Minute Walk Test Conducted Using 15‐Meter and 30‐Meter Walkways.” Topics in Stroke Rehabilitation 27, no. 4: 251–261. 10.1080/10749357.2019.1691815. 31752634

[pri70201-bib-0011] Di Thommazo‐Luporini, L. , S. P. Jürgensen , V. Castello‐Simões , A. M. Catai , R. Arena , and A. Borghi‐Silva . 2012. “Metabolic and Clinical Comparative Analysis of Treadmill Six‐Minute Walking Test and Cardiopulmonary Exercise Testing in Obese and Eutrophic Women.” Revista Brasileira de Fisioterapia 16, no. 6: 469–478. 10.1590/S1413-35552012005000036. 22832701

[pri70201-bib-0012] Fell, B. , S. Hanekom , and M. Heine . 2022. “A Modified six‐minute Walk Test (6MWT) for low‐resource settings‐a cross‐sectional Study.” Heart & Lung: Journal of Critical Care 52: 117–122. 10.1016/j.hrtlng.2021.12.008. 35007887

[pri70201-bib-0013] Flansbjer, U.‐B. , A. M. Holmbäck , D. Downham , C. Patten , and J. Lexell . 2005. “Reliability of Gait Performance Tests in Men and Women With Hemiparesis After Stroke.” Journal of Rehabilitation Medicine 37, no. 2: 75–82. 10.1080/16501970410017215. 15788341

[pri70201-bib-0014] Gagnier, J. J. , J. Lai , L. B. Mokkink , and C. B. Terwee . 2021. “COSMIN Reporting Guideline for Studies on Measurement Properties of Patient‐Reported Outcome Measures.” Quality of Life Research 30, no. 8: 2197–2218. 10.1007/s11136-021-02822-4. 33818733

[pri70201-bib-0015] Green, J. , A. Forster , and J. Young . 2002. “Reliability of Gait Speed Measured by a Timed Walking Test in Patients One Year After Stroke.” Clinical Rehabilitation 16, no. 3: 306–314. 10.1191/0269215502cr495oa. 12017517

[pri70201-bib-0016] Hebert, D. , M. P. Lindsay , A. McIntyre , et al. 2016. “Canadian Stroke Best Practice Recommendations: Stroke Rehabilitation Practice Guidelines, Update 2015.” International Journal of Stroke 11, no. 4: 459–484. 10.1177/1747493016643553. 27079654

[pri70201-bib-0017] Huang, S. L. , C. L. Hsieh , R. M. Wu , C. H. Tai , C. H. Lin , and W. S. Lu . 2011. “Minimal Detectable Change of the Timed ‘Up and Go’ Test and the Dynamic Gait Index in People With Parkinson Disease.” Physical Therapy 91, no. 1: 114–121. 10.2522/ptj.20090126. 20947672

[pri70201-bib-0018] Kossi, O. , B. Bonnechère , M. Agbetou , et al. 2024. “Relationships Between Cardiorespiratory Fitness, Physical Activity Practices, and Functional Outcomes One Year Post‐Stroke in Northern Benin: A Case‐Control Study.” Topics in Stroke Rehabilitation 31, no. 1: 104–115. 10.1080/10749357.2023.2207286. 37120850

[pri70201-bib-0019] Larsson, P. , E. Edvardsen , C. L. Gay , M. Ursin , U. Mack , and A. Lerdal . 2024. “Cardiorespiratory Fitness, Physical Activity, and Fatigue Three Months After First‐Ever Ischemic Stroke.” Topics in Stroke Rehabilitation 31, no. 8: 817–827. 10.1080/10749357.2024.2333191. 38533786

[pri70201-bib-0020] MacKay‐Lyons, M. , S. A. Billinger , J. J. Eng , et al. 2020. “Aerobic Exercise Recommendations to Optimize Best Practices in Care After Stroke: AEROBICS 2019 Update.” Physical Therapy 100, no. 1: 149–156. 10.1093/ptj/pzz153. 31596465 PMC8204880

[pri70201-bib-0021] Mokkink, L. B. , C. B. Terwee , D. L. Knol , et al. 2010. “The COSMIN Checklist for Evaluating the Methodological Quality of Studies on Measurement Properties: A Clarification of Its Content.” BMC Medical Research Methodology 10, no. 1: 22. 10.1186/1471-2288-10-22. 20298572 PMC2848183

[pri70201-bib-0022] Munro, B. 2005. Statistical Methods for Health Care Research. 5th ed. Lippincott Williams and Wilkins.

[pri70201-bib-0023] Nascimento, L. R. , A. Boening , A. Galli , J. C. Polese , and L. Ada . 2021. “Treadmill Walking Improves Walking Speed and Distance in Ambulatory People After Stroke and is Not Inferior to Overground Walking: A Systematic Review.” Journal of Physiotherapy 67, no. 2: 95–104. 10.1016/j.jphys.2021.02.014. 33744188

[pri70201-bib-0024] Nascimento, L. R. , L. C. G. Caetano , D. C. M. A. Freitas , T. M. Morais , J. C. Polese , and L. F. Teixeira‐Salmela . 2012. “Different Instructions During the Ten‐Meter Walking Test Determined Significant Increases in Maximum Gait Speed in Individuals With Chronic Hemiparesis.” Revista Brasileira de Fisioterapia 16, no. 2: 122–127. 10.1590/s1413-35552012005000008. 22378478

[pri70201-bib-0025] Oyake, K. , T. Yamaguchi , C. Oda , et al. 2017. “Unilateral Arm Crank Exercise Test for Assessing Cardiorespiratory Fitness in Individuals With Hemiparetic Stroke.” BioMed Research International 2017: 6862041–10. 10.1155/2017/6862041. PMC5804117 29457034

[pri70201-bib-0026] Pessoa, B. V. , J. F. Arcuri , I. G. Labadessa , J. N. F. Costa , A. C. Sentanin , and V. A. P. Di Lorenzo . 2014. “Validity of the Six‐Minute Step Test of Free Cadence in Patients With Chronic Obstructive Pulmonary Disease.” Brazilian Journal of Physical Therapy 18, no. 3: 228–236. 10.1590/BJPT-RBF.2014.0041. 25003275 PMC4183495

[pri70201-bib-0027] Polese, J. C. , L. Ada , and L. F. Teixeira‐Salmela . 2018. “Relationship Between Oxygen Cost of Walking and Level of Walking Disability After Stroke: An Experimental Study.” Physiotherapy Research International 23, no. 1: e1688. 10.1002/pri.1688. 28671315

[pri70201-bib-0028] Portney, L. G. 2020. Foundations of Clinical Research: Applications to Evidence‐Based Practice. 4th ed., 486–508 F. A. Davis Company.

[pri70201-bib-0029] Quintino, L. F. , L. T. Aguiar , S. A. F. de Brito , A. S. Pereira , L. F. Teixeira‐Salmela , and C. D. C. de Morais Faria . 2021. “Reliability and Validity of the Incremental Shuttle Walking Test in Individuals After Stroke.” Topics in Stroke Rehabilitation 28, no. 5: 331–339. 10.1080/10749357.2020.1818481. 32924882

[pri70201-bib-0030] Richards, C. L. , F. Malouin , and C. Dean . 1999. “Gait in Stroke: Assessment and Rehabilitation.” Clinics in Geriatric Medicine 15, no. 4: 833–855. 10.1016/S0749-0690(18)30034-X. 10499938

[pri70201-bib-0031] Smith, A. C. , D. H. Saunders , and G. Mead . 2012. “Cardiorespiratory Fitness After Stroke: A Systematic Review.” International Journal of Stroke 7, no. 6: 499–510. 10.1111/j.1747-4949.2012.00791.x. 22568786

[pri70201-bib-0032] Souza, A. C. de , N. M. C. Alexandre , E. de B. Guirardello , and N. M. C. Alexandre . 2017. “Psychometric Properties in Instruments Evaluation of Reliability and Validity.” Epidemiologia e Serviços de Saúde 26, no. 3: 649–659. 10.5123/S1679-49742017000300022. 28977189

[pri70201-bib-0033] Sullivan, J. E. , B. E. Crowner , P. M. Kluding , et al. 2013. “Outcome Measures for Individuals With Stroke: Process and Recommendations From the American Physical Therapy Association Neurology Section Task Force.” Physical Therapy 93, no. 10: 1383–1396. 10.2522/ptj.20120492. 23704035

[pri70201-bib-0034] Tseng, B. Y. , B. J. Gajewski , and P. M. Kluding . 2010. “Reliability, Responsiveness, and Validity of the Visual Analog Fatigue Scale to Measure Exertion Fatigue in People With Chronic Stroke: A Preliminary Study.” Stroke Research and Treatment 2010: 1–7. 10.4061/2010/412964. PMC2911654 20700421

[pri70201-bib-0035] Tyson, S. , and L. Connell . 2009. “The Psychometric Properties and Clinical Utility of Measures of Walking and Mobility in Neurological Conditions: A Systematic Review.” Clinical Rehabilitation 23, no. 11: 1018–1033. 10.1177/0269215509339004. 19786420

